# Supplementary material for: Relationship between COVID-19 and ICU-Acquired Bloodstream Infections Related to Multidrug-Resistant Bacteria
Source: Antibiotics (Basel). 2023 Jun 25;12(7):1105. doi: 10.3390/antibiotics12071105 (PMC10376231; doi:10.3390/antibiotics12071105)
Supplement: Supplementary file 1 [file antibiotics-12-01105-s001.zip › antibiotics-2370401-supplementary.pdf]

Table S1. Relationship between COVID-19 and ICU-acquired BSI related to MDRB by Fine et Gray Model

	COVID(-)	COVID(+)	Unadjusted analysis		Adjusted analysis	
	(n=823)	(n=497)	sHR (95%CI)	P-Value	sHR (95%CI)	P-Value
**MDRB**	14/823 (1.7)	36/497 (7.2)	4.34 (2.33 to 8.06)	<0.001	5.76 (2.82 to 11.73)	<0.001
0 to 14 days[1]	8/823 (1.0)	8/497 (1.6)	1.66 (0.62 to 4.42)	0.18	2.20 (0.81 to 5.94)	0.063
15 to 30 days	6/137 (4.2)	28/190 (14.8)	7.93 (3.27 to 19.20)	<0.001	10.53 (3.84 to 28.84)	<0.001

Figure S1.

60-day cumulative incidence of A) ICU-mortality, B) Extubation Alive (mechanical ventilation duration), and C) ICU discharge alive (length of ICU stay) according to disease study groups.

Cumulative incidences were estimated using Kalbfleisch and Prentice method by considering for A), ICU mortality as event of interest and ICU-discharge alive as competing event, for B), extubation alive as event of interest and death under mechanical ventilation as competing event, and C) ICU discharge alive as event of interest and ICU mortality as competing event.

A)

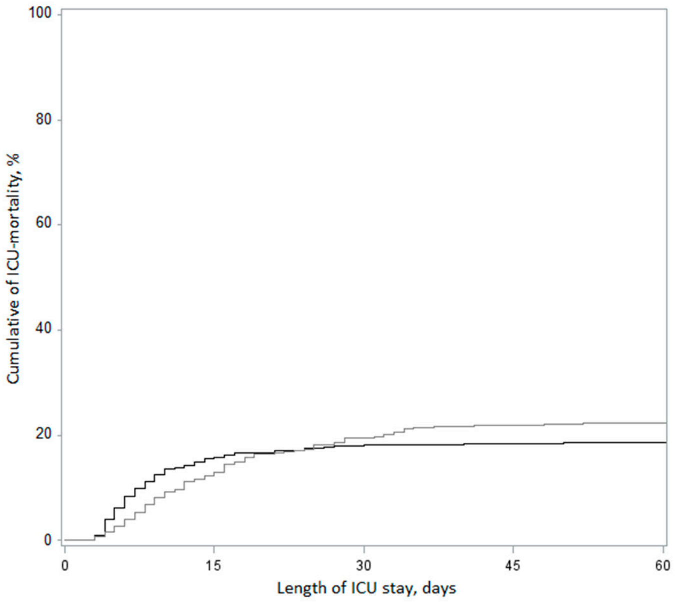

		0	15	30	45	60
Covid (-)	At risk	823	131	41	23	0
	Death	0	131	149	151	152
	ICU discharge Alive	0	561	632	648	656
	Censored	0	0	0	0	14
Covid (+)	At risk	497	181	69	34	0
	Death	0	65	97	108	111
	ICU discharge Alive	0	251	330	354	369
	Censored	0	0	0	0	16

B)

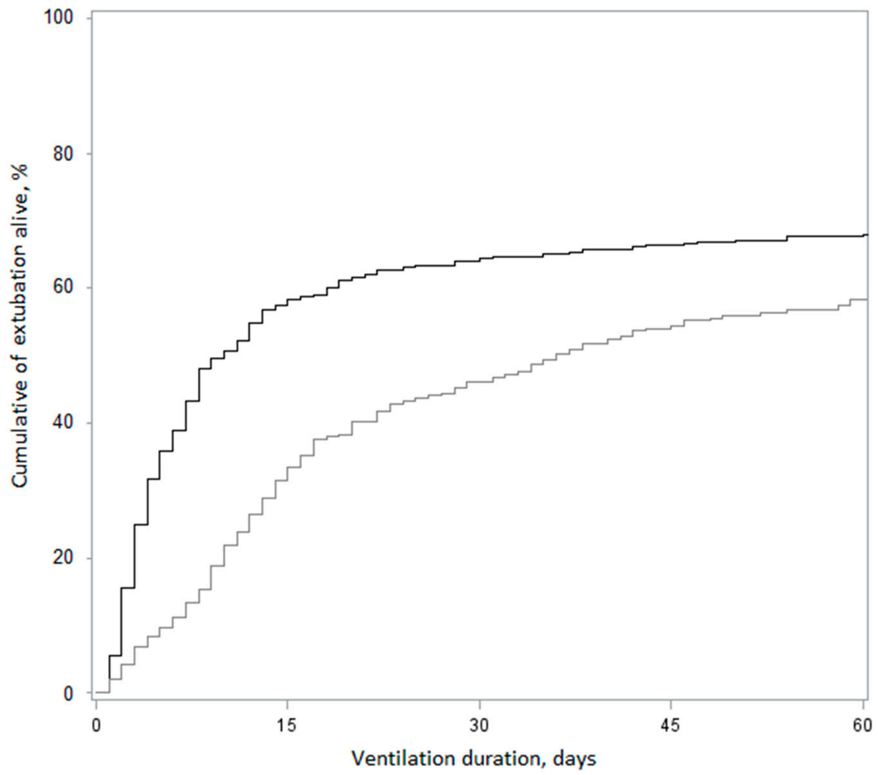

		0	15	30	45	60
Covid (-)	At risk	398	59	19	9	0
	extubation Alive	0	232	256	264	270
	Death	0	107	123	125	125
	Censored	0	0	0	0	3
Covid (+)	At risk	261	117	51	22	0
	extubation Alive	0	87	120	142	152
	Death	0	57	123	125	125
	Censored	0	0	0	0	3

C)

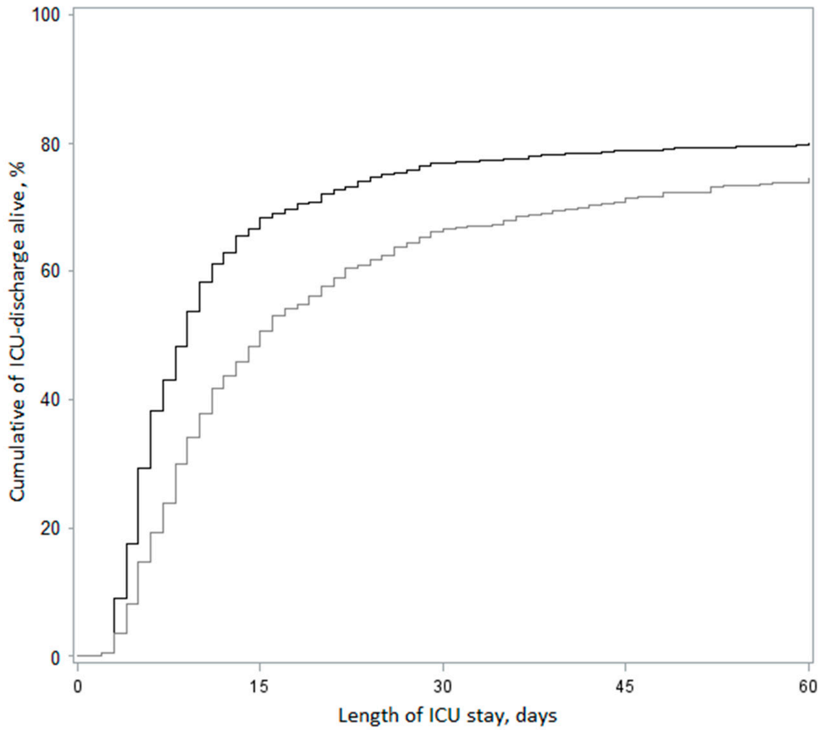

		0	15	30	45	60
Covid (-)	At risk	823	131	41	23	0
	ICU discharge Alive	0	561	632	648	656
	Death	0	131	149	151	152
	Censored	0	0	0	0	14
Covid (+)	At risk	497	181	69	34	0
	ICU discharge Alive	0	251	330	354	369
	Death	0	65	97	108	111
	Censored	0	0	0	0	16
